# Postpandemic Influenza A/H1N1pdm09 is still Causing Severe Perinatal Complications

**DOI:** 10.4084/MJHID.2015.007

**Published:** 2015-01-01

**Authors:** Hein Bogers, Dominique Bos, Sam Schoenmakers, Johannes J Duvekot

**Affiliations:** Erasmus MC, University Medical Centre Rotterdam, Department of Obstetrics and Gynaecology, Division of Obstetrics and Prenatal Medicine, University Medical Centre Rotterdam.

## Abstract

Although influenza A/H1N1pdm09 is not causing a pandemic anymore, we recently observed two critically ill pregnant women infected by this virus. We present these cases to illustrate the possible severe complications of an – at that moment – seasonal influenza in pregnancy. We discuss the epidemiological differences between the pandemic and post pandemic phase and try to explain the high virulence of influenza A/H1N1pdm09 -infections in pregnancy by discussing insights in immunology during pregnancy. We conclude that although influenza A/H1N1pdm09 is in the post pandemic phase, infection by this influenza virus still needs to be considered in pregnant women with progressive respiratory dysfunction.

## Introduction

During the influenza A/H1N1pdm09 or swine flu pandemic, pregnant women appeared to be at increased risk for admission to an intensive care unit (ICU) because of complications of this viral disease. Although the World Health Organization has announced the disease to be postpandemic since August 2010, several mutations capable of causing severe illness have been isolated afterwards. After this pandemic phase, we admitted several pregnant women who were severely affected by influenza A/H1N1pdm09. In this article, we would like to draw attention to the possible severe complications of an - at that moment - seasonal influenza in pregnancy and try to explain its immunological origins. We hope to contribute to a greater awareness of influenza-related morbidity during pregnancy to prepare for future influenza pandemics.

## Case Reports

A 28-year-old sub-Saharan African multiparous woman with a history of recent syphilis and human immunodeficiency virus (HIV) infection was treated with highly active antiretroviral treatment (HAART). Viral load was undetectable. She had not received influenza vaccination during the pandemic in 2009, nor in the postpandemic phase. At a gestational age (GA) of 37 weeks, she was admitted to our hospital because of contractions in combination with tachypnoea and fever. Because of foetal distress, an emergency Caesarean section was performed, and a healthy female neonate was born. After delivery, maternal condition deteriorated and a computed tomography (CT) scan revealed multiple pulmonary infiltrations. A throat swab using a polymerase chain reaction (PCR) was only positive for influenza A/H1N1pdm09. She was treated with oseltamivir orally. Four days later she was transferred to the intensive care unit (ICU) because of respiratory failure needing intubation. Not earlier than after two months the PCR became negative, and she was weaned from mechanical ventilation. A CT scan revealed afterwards severely affected lungs with focal fibrosis, bronchiectasis and bronchial wall thickening.

The second patient is also a 28-year-old sub-Saharan nulliparous woman. Apart from a malaria infection more than ten years ago, she was in good health and accordingly had not received influenza vaccination. At a GA of 21 weeks, she reported with a cough and fever and was subsequently admitted. Chest radiograph revealed multiple infiltrations. PCR on a throat swab was only positive for influenza A/H1N1pdm09 and treatment with oseltamivir was started. Despite the treatment, her clinical condition worsened and mechanical ventilation was needed. Subsequently, foetal demise was diagnosed at a GA of 24 weeks, and labour was induced in an effort to improve her respiratory condition. The stillborn neonate had no visible anomalies. Although the PCR for influenza A/H1N1pdm09 became negative, she could not be weaned from mechanical ventilation. After three weeks, the steroid treatment was started under suspicion of a cryptogenic organising pneumonia, resulting in a dramatic improvement in respiratory function resulting in a full recovery.

## Discussion

### Pandemic (H1N1) 2009 and pregnancy

To date there seems to be no major difference in virulence between influenza A/H1N1pdm09 and seasonal influenza strains in the general population.^1^ However, pregnancy is a recognised risk factor for a more severe and complicated outcome from influenza A/H1N1pdm09. Shiley et al. observed in 16% of the influenza A/H1N1pdm09 infections a concomitant pregnancy in contrast to 1% of the seasonal influenza infections (p<0.001).^2^ Mosby et al. showed that pregnancy increased the odds of being admitted to the hospital or ICU.^3^ Of the influenza A/H1N1pdm09 -infected women of reproductive age admitted to the ICU, 21.4% was pregnant.^4^ Furthermore, pregnant women were disproportionally represented among the influenza A/H1N1pdm09 deaths.^3^ Ellington et al. wrote a case series of influenza A/H1N1pdm09 -infected critically ill pregnant women. Of these women, 42 had to be mechanically ventilated, and 15 of them died (36%).^5^

Regarding perinatal morbidity, a large cohort study by Pierce et al. showed this was up to six-fold increased in children born to influenza A/H1N1pdm09 -infected women, as compared to uninfected women.^6^ This was primarily caused by an increased stillbirth rate (3 % v. 1 %, p = 0.001). Preterm birth was more observed as well, and those women also had higher odds of being admitted to the ICU (54% v. 12%, p<0.001).

Our patients were immigrants. In the only study on this subject, the incidence of influenza infection in the native and immigrant population was investigated during the pandemic in Spain by Esteban-Vasallo et al. A trend was seen towards more severe influenza cases in immigrant women of childbearing age compared to native women (21.3% v.12.3%, p = 0.06).^7^

### Pregnancy as risk factor

There are studies comparing severity of disease in pregnant versus non-pregnant mice. Chan et al. showed that A/H1N1pdm09 demonstrated in pregnant mice infection with influenza damage mainly confined to the lungs, with no signs of extrapulmonary dissemination.^8^ The pregnant mice exhibited more severe pneumonia, damaged lung epithelium and higher mortality as compared with the non-pregnant mice. The lung damage was associated with an increased pro-inflammatory and pro-Th-2 cytokine profile, whereas the Th-1 (antiviral) immune response was similar to significant lower in the pregnant group. Other groups also reported a slower innate immune response to infection with influenza A/H1N1pdm09 in pregnant mice^9^ and inhibition of cytotoxic natural killer cells,^10^ which would allow a higher replication rate of the virus. A subsequent increased pulmonary infiltration by macrophages and neutrophils with high production of nitric oxide damages the pulmonary epithelium,^8^,^9^ which already has a slower level of regeneration due to infection. This can lead to increased pulmonary permeability, thus compromising the respiratory capacity further. Moreover, with ongoing pregnancy a more decreased pulmonary capacity can be observed. As GA progresses, the enlarging uterus causes an increase in abdominal pressure, resulting in a decreased chest wall compliance and diaphragmatic elevation. Furthermore, lower levels of respiratory maternal immunoglobulins G (IgGs) after the initial contact with influenza A/H1N1pdm09 are reported.^10^ It appears that during pregnancy maternal IgG-antibodies become trapped in the placenta to protect the developing foetus, while making the IgG-antibodies unavailable for the maternal immune defence.^8^,^9^ IgG-antibodies play a major role in the lower respiratory tract,^11^ further contributing to the fulminant course of influenza A/H1N1pdm09 -infection in pregnancy.

### Postpandemic influenza A/H1N1pdm09

Our two patients were admitted after the Dutch pandemic. So far, no studies described the features of this post pandemic wave in pregnant patients. There have been few reports describing the differences between the pandemic and post pandemic influenza A/H1N1pdm09 -infection in non-pregnant patients. These reports all mention a marked increase in severity during the post pandemic phase,^12^–^15^ which could be due to mutations of the virus. The rate ratio for hospitalisation was 2.6 (95% CI 2.3–2.9) and patients appeared to be older during the post pandemic (median 35 years) than during the pandemic phase (median 20 years) (p<0.0001).^12^ Time from onset of illness to administration of antiviral therapy and length of stay was also longer, and need for mechanical ventilation and ICU admission were significantly higher during the post pandemic period.^14^

### Future pandemics

Investigating influenza pandemics might help to prepare for future pandemics, but influenza will remain a threat to mankind due to continuing mutations of the influenza A-specific antigens. Although influenza A/H1N1pdm09 is in its post pandemic phase, severe illness remains possible. In all pregnant women with respiratory problems, infection with influenza A/H1N1pdm09 always needs to be considered. Early diagnosis and treatment may reduce the risk of severe illness.^16^ Vaccination is still highly recommended even after the pandemic influenza A/H1N1pdm09 phase.^17^
